# Sustainable Ornamental Fish Aquaculture: The Implication of Microbial Feed Additives

**DOI:** 10.3390/ani13101583

**Published:** 2023-05-09

**Authors:** Seyed Hossein Hoseinifar, Francesca Maradonna, Mehwish Faheem, Ramasamy Harikrishnan, Gunapathy Devi, Einar Ringø, Hien Van Doan, Ghasem Ashouri, Giorgia Gioacchini, Oliana Carnevali

**Affiliations:** 1 Department of Fisheries, Faculty of Fisheries and Environmental Sciences, Gorgan University of Agricultural Sciences and Natural Resources, Gorgan 49189-43464, Iran; 2 Department of Life and Environmental Sciences, Polytechnic University of Marche, 60131 Ancona, Italy; f.maradonna@univpm.it (F.M.); giorgia.gioacchini@staff.univpm.it (G.G.); 3 Department of Zoology, Government College University, Lahore 54000, Pakistan; 4 Department of Zoology, Pachaiyappa’s College for Men, Kanchipuram 631501, Tamil Nadu, India; 5 Department of Zoology, Nehru Memorial College, Puthanampatti 621007, Tamil Nadu, India; 6 Norwegian College of Fishery Science, Faculty of Bioscience, Fisheries and Economics, UiT The Arctic University of Norway, N9019 Tromsø, Norway; 7 Department of Animal and Aquatic Sciences, Faculty of Agriculture, Chiang Mai University, Chiang Mai 50200, Thailand

**Keywords:** probiotic, prebiotic, synbiotic, fish disease, immune system, reproduction, growth, feed additives

## Abstract

**Simple Summary:**

In recent decades, trade of ornamental fish has significantly increased. A rise in demand was observed, especially from top importing countries that contributed majorly to the growth of the market. The destructive fishing methods caused a severe impairment of natural and environmental resources. Thus, there is a need to improve ornamental fish aquaculture, increasing the number of cultured species and limiting wild fish handling and transport stress losses. In this light, the use of microbial feed additives such as probiotics, prebiotics, and synbiotics, could help in improving the immune system and growth as well as increasing reproductive performance in captivity-bred species.

**Abstract:**

Ornamental fish trade represents an important economic sector with an export turnover that reached approximately 5 billion US dollars in 2018. Despite its high economic importance, this sector does not receive much attention. Ornamental fish husbandry still faces many challenges and losses caused by transport stress and handling and outbreak of diseases are still to be improved. This review will provide insights on ornamental fish diseases along with the measures used to avoid or limit their onset. Moreover, this review will discuss the role of different natural and sustainable microbial feed additives, particularly probiotics, prebiotics, and synbiotics on the health, reduction in transport stress, growth, and reproduction of farmed ornamental fish. Most importantly, this review aims to fill the informational gaps existing in advanced and sustainable practices in the ornamental fish production.

## 1. Introduction

Ornamental fishes, due to their different and brilliant colors, shapes and behavior, are often referred to as living jewels and are kept in aquaria or garden pools for their beauty as well as entertainment. Ornamental fishes thank to their different and brilliant colors, shape and behavior, are often referred as living jewels and are kept in aquaria or garden pools for entertaining and fancy thus resulting, together with photography, among the most important hobbies worldwide. Owning and photography of ornamental fishes are popular hobbies worldwide. There is an increase in people who are more inclined towards purchasing attractive fish species for decorative purposes, citing their alluring features and differing characteristics. This has fueled the exponential growth of the ornamental fish market [1]. Moreover, the latest technological advancements in the industry, such as pet cameras and automatic filters, have further augmented the desire to adopt pets. 

Therefore, in recent decades, the global trade of these “pets” has rapidly increased. In a recent review dealing with marine ornamental fish larviculture, Chen et al. [2] stated “each year, it is estimated that more than 20 million marine ornamental fish are collected from the wild and sold to over 2 million aquarium hobbyists world-wide”, which indicates the dire need to expand this industry by increasing the number of new ornamental species [2].

Ornamental fish breeding started in China more than 1000 years ago when goldfish, a freshwater fish, was domesticated. Then, in the 1930s, in Sri Lanka, marine trade started as a result of the export of coral reefs for aquariums [3,4]. However, the fish industry only gained real economic importance from the 1950s onwards. Although the industry has expanded considerably, the production of fish declined in the late 1990s. 

Nowadays, more than 7000 aquatic species are reared and marketed as ornamental fish. Of them, approximately 5000 are freshwater- and 1800 are marine species [2,5,6,7]. In contrast to marine ones, the majority of freshwater specimens are produced in captivity [8]. More than 120 countries are involved in the ornamental fish industry, in their import/export, led by Asian and developing countries, which produce approximately 60% of ornamental fish [9], with a global trade worth approximately of USD 15–30 billion each year [4,9].

Very recently, some comprehensive reviews on ornamental fish culture have been published [2,7,10], detailing the global interest in ornamental fish. To meet this increasing demand, the introduction of innovative and sustainable rearing practices could represent an improvement in the aquaculture sector, reducing the depletion of natural resources in many developing countries. 

Unfortunately, none of these review articles deepened the understanding of the role of microbial feed additives on growth performance, immune response, fecundity, stress resistance and improvement of water quality, in ornamental fish farming. Thus, the present review will collect all the available knowledge regarding these lesser-known aspects of fundamental importance to realize a sustainable ornamental fish aquaculture.

## 2. Economic Aspects of Ornamental Fish Trade 

The ornamental fish market was valued at USD 5.4 billion in 2021 and is anticipated to expand at a compound annual growth rate (CAGR) of 8.5% from 2022 to 2030. The tropical freshwater fish segment dominates the market and accounted for the largest revenue share of 51.7% in 2021. Among most popular tropical freshwater fish for beginners are guppy (*Poecilia reticulata*), molly (*Poecilia sphenops*), and zebrafish (*Danio rerio*); most of them are quite inexpensive and usually priced between USD 1 and 6. Nearly 15% of the total traded fish consists of marine ornamental species, with their attractive colors and attention-grabbing behavior [11], largely derived from wild catches [12].

Approximately 99% of the market is kept by hobbyists and less than 1% by public aquaria and research institutes. It has been estimated that more than 1.5 million people are involved in this sector and there are over 3.5 million hobbyists worldwide involved in this trade. The sector has provided employment opportunities, alleviated poverty, and contributed to national income by enhancing foreign exchange earnings [13].

World exports of ornamental fish increased steadily from USD 177.7 million to a peak of USD 364.9 million in 2011, before declining slightly to USD 347.5 million in 2014 [14].

**Figure 1:**
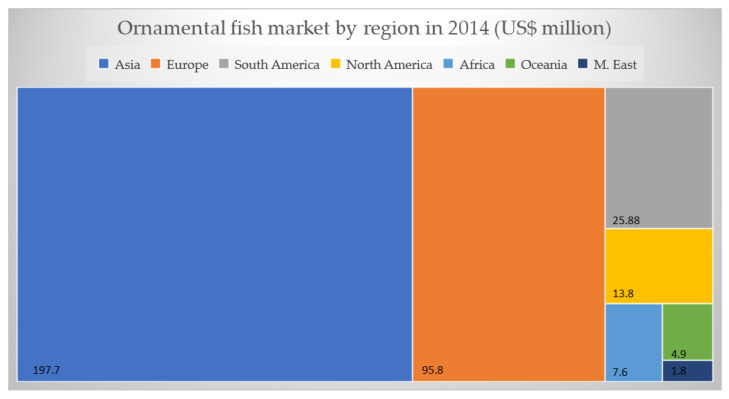
Export share of major markets by region, 2014 (in USD million).

**Figure 2:**
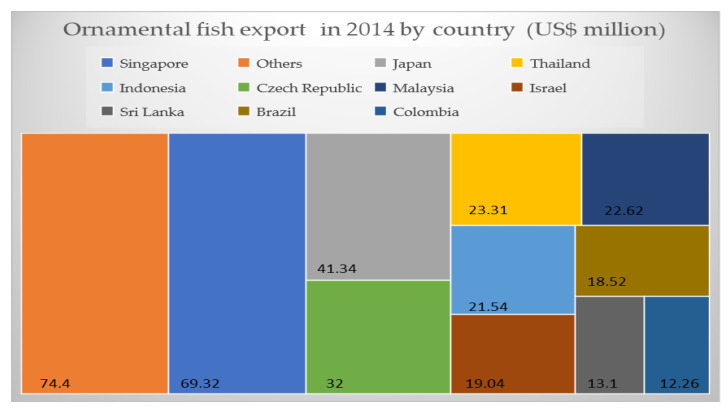
Export share of major markets by country, 2014 (in USD million).

In 2014, Asian countries accounted for more than 50% of the trade, and export was valued at USD 130 million. Europe accounted for 27.6% of the total market (valued at USD 95.8 million) followed by South America (7.5%), North America (3.98%), Africa (2.2%), Oceania (1.4%) and the Middle East (0.5%) (Figure 1). Singapore was the top ornamental fish exporter (20% of the total) and the turnover was valued at USD 69.32 million [14] (Figure 2). Japan was the leader in koi carp production, thus the second most important exporting country (USD 41.34 million). Starting from 2000, for many years, the USA had the world’s largest ornamental fish market, but imports declined from USD 60 million in 2000 to USD 42.9 million in 2014 [14]. In Europe, the UK remains the largest importer of ornamental fish (USD 29.5 million) [15]. 

## 3. Effects of Microbial Feed Additives on Ornamental Fish Health

Disease outbreak is the major problem in ornamental fish production, causing a substantial economic loss of approximately 400 million US dollars [16]. Diseases can be either of parasitic, bacterial, viral or fungal origins and common symptoms include dropsy, ulcers, fin and tail rot, constipation, swim bladder inflammation, clamped fins, pop eyes, flip over disease, skin flukes, and cloudy eye. The resulting losses negatively affect the financial and socioeconomic status of the ornamental fish farming community. Table 1, Table 2, Table 3 and Table 4 list the most common parasitic, bacterial, viral, and fungal diseases identified so far.

Commercial aquaculture practices commonly used to prevent or heal damage in fish intended for human consumption, can represent a good starting point to reduce losses The aquaculture industry still represent a major area of antibiotic misuse [17] and so far, oxytetracycline hydrochloride and kanamycin have positively resolved a wide spectrum of fish bacterial diseases, including furunculosis, aeromonosis, pseudomonosis, lactococcosis, and vibriosis, being administered via feed, bath treatment or injection [18]. Excessive and unregulated use of antibiotics in aquaculture, as well as in the ornamental fish industry, has led to the development of gut antibiotic-resistant bacteria [19] and antimicrobial-resistant pathogens [20], subsequently affecting the immune system of fish.

Starting from the regulation of antibiotic use, researchers are now focused on considering and discussing valid alternatives and promising functional feed additives (vaccination, bacteriophages, quorum quenching, probiotics and prebiotics, chicken egg yolk antibody and medicinal plant derivative) that could also be successfully applied in ornamental fish culture [21].

Lilly and Stillwell [22] first proposed the term probiotics “to be used for substances that favors the growth of microorganisms”. Since then, several definitions have been proposed and the most common is “*live microorganisms that when administrated in adequate amounts*, *confer a health benefit to the host*”. proposed by the World Health Organization (WHO). Kozasa [23] was the first researcher who used probiotics in aquaculture and the very first review article on probiotics was published in 1998 [24]; since then, several reviews have been published [25,26,27,28,29,30,31,32,33]. Live bacteria as well as inactivated bacteria and spore formers have been used as probiotics in aquaculture [34]. Among microbial fish additives, lactic acid bacteria (LAB) and *Bacillus* probiotics are the most used; however, *Aeromonas*, *Alteromonas*, *Arthrobacter*, *Bifidobacterium*, *Clostridium*, *Paenibacillus*, *Phaeobacter*, *Pseudoalteromonas*, *Pseudomonas*, *Rhodosporidium*, *Roseobacter*, *Streptomyces*, *Vibrio*, microalgae (*Tetraselmis*), and yeast (*Debaryomyces*, *Phaffia*, and *Saccharomyces*) are also beneficial [33]. Probiotics can be administered via feed supplementation (single or in mixture) or dissolved in water. 

Starting from the beginning of the 1980s, probiotics were used in aquaculture practices aiming at controlling bacterial infections and improving water quality. In teleost species, increasing evidence confirmed that probiotics can increase lipid and glucose levels [35,36,37], bone metabolism [38], microbiome composition [39], stress tolerance [40,41], and reproductive performance [26,42,43,44,45]. Nowadays, several commercial probiotics containing *Bacillus* sp., *Lactobacillus* sp., *Enterococcus* sp., *Carnobacterium* sp., and yeast *Saccharomyces cerevisiae* are used with strict safety measurements and careful management recommendations, which guarantee beneficial effects on production and health [46]. 

Table 5 represents the possible effects of microbial feed additive on ornamental fish health, welfare and reproduction.

LAB, a variety of Gram-positive bacteria, are the main microorganisms that ferment plants, vegetables, meats, fish, and dairy products in the intestine [83,84]. LAB are also commonly used to produce various compounds, such as small organic acids, vitamins, and biological peptides [85,86,87]. Within LAB, *L. acidophilus* is among the most industrially utilized strain in the manufacture of dairy products and dietary supplements [88,89]. Given that LAB may supply several organic molecules via various metabolic processes, these microbes could be utilized as valuable and specific sources of a wide range of enzymes with novel properties [90,91]. 

Production of Siamese fighting fish (*Betta splendens*) represents a great example of good practice using LAB. This species provides a great income among exported ornamental fish in Thailand. A study reported that a diet supplemented with *L. plantarum* (KKU CRIT5) exerted no significant improvement of digestive enzymes compared to control, with decreased protein depositions in body and muscle, suggesting that additional trials could be set up in this species in order to verify the effects of this probiotic focusing on dose and time of administration [47]. In *Xiphophorus helleri*, a dietary supplementation of *L. acidophilus* positively modulated mucosal immune parameters, e.g., skin mucus protein and alkaline phosphatase levels. In addition, the response to salinity stress was improved, clearly indicating a better healthy condition of the fish, as also supported by the modulation of intestinal microbiota towards beneficial LAB [48]. Surprisingly, in the same species, the administration of a commercial probiotic formulation containing two *Lactobacillus* species, two *Bacillus species*, *Streptococcus faecium*, and *S. cereviasiae* did not confer better tolerance against bacterial challenges [49]. Similar results were observed in goldfish, *Carassius auratus*, where the administration of the same commercial probiotic or a mixture of *Lactobacillus* sp. and *Bacillus* sp. had no significant effect on resistance against *Pseudomonas fluorescens* infection [49]. Additionally, a study on goldfish reported that when a 40% protein diet was supplemented with different concentrations of three commercial *Lactobacillus* probiotic-based formulations, an increase in protein and a decrease in fat content were observed. This suggested that these probiotics could improve the nutritional properties of the fillet [54], which is an important factor, especially for species intended for human consumption. Porthole livebearer (*Poecilopsis gracilis*), when fed with *Artemia* nauplii enriched with *L. casei*, showed increased production of skin mucus and a faster recovery after the air-dive/stress test, suggesting that this probiotic could be helpful in obtaining healthier organism for the ornamental fish market [56].

*B. coagulans* and *B. mesentericus*, used to enrich *Thermocyclops decipiens* cultures and administered via diet to *Puntius conchonius* post larvae, significantly changed gut microflora composition. Microbiota analysis further revealed that *B. coagulans* poorly adheres to the gut with respect to *B. mesentericus* [57].

The commercial mixture containing *B. subtilis*, *B. licheniformes*, *L. acidophilus*, and *S. cerevisiae* was beneficial in fish facing extreme conditions including handling and transport stress. Based on these results, these probiotic strains have been largely used in the trade of Cardinal tetra, *Paracheirodon axelrodi* [75] and the marbled hatchet fish, *Carnegiella strigata* [74], which showed a decrease in stress levels and related metabolite secretion. In these trials, a sensible improvement of water quality was also described. 

Probiotics (*Bacillus* sp., *Lactobacillus* sp., and their mixtures) were used in the giant gourami, *Osphronemus goramy*, to produce higher-quality fish and to reduce the risk of diseases outbreak. Fish exposed to a mixture of *Bacillus* sp. and *Lactobacillus* sp. via water presented a higher survival rate. These species also improved the quality of the rearing water [58]. 

*L. fermentum* (KT183369) and *B. subtilis *sp.* inaquasporium* (KR816099) isolated from coconut were used in a feeding trial with the black molly, *Poecilia sphenops*. At the end, their adhesive properties towards the host cells were found, which led to the speculation that both strains could be used against *Vibrio parahaemolyticus*. The ability to fight *V. parahaemolyticus* was demonstrated by a challenge test. In addition, *L. fermentum* displayed a higher capacity to colonize the gut, suggesting that could be an excellent feed additive for ornamental aquaculture species [59,60]. An additional challenge test against *V. anguillarum* was set up using *B. pumilus* RI06-95Sm and results showed its ability to colonize the molly’s gut, reverse the negative impacts of antibiotic treatment and decrease the mortality rate [61]. 

Three different probiotic strains—*L. rhamnosus*, *B. coagulans*, and *B. mesentericus*—were administered separately to zebrafish, *D. rerio*, through enrichment of artemia and compared with control fish fed with unenriched artemia. The positive effect of probiotic administration was demonstrated by the decrease of the number of gut pathogenic bacteria in all experimental groups compared to control fish. One week after the end of the trial, gut microbiota was significantly colonized by *L. rhamnosus*, suggesting artemia as an effective means for probiotic delivery [62]. *L. rhamnosus* administration via water to zebrafish larvae significantly accelerated skeletogenesis acting on lipid and vitamin D metabolism [63] and positively modulated the expression of genes responsible for bone formation [38]. A positive modulation of signal involved in lipid and vitamin D metabolism was also observed in clownfish, *Amphiprion ocellaris* [71]. Positive outcomes were also observed using caudal fin regeneration as a process to investigate the effects of a mix of *B. subtilis*, *B. licheniformis*, *B. coagulans*, and *L. acidophilus* plus the yeast *S. cerevisiae*. In this study, evidence regarding the treatmentability to promote osteoblast differentiation, suppress osteoclast activity and modulate phosphate homeostasis was provided, strongly promoting its use to support bone homeostasis and reduce deformity [92].

Zebrafish also resulted an excellent experimental model to demonstrate the positive role of *L. rhamnosus* IMC 501 on fish welfare: an increase of genes involved in immunity was recorded in intestinal tissue, while in the liver, a downregulation of stress and apoptotic-related biomarkers was documented, suggesting that administration of probiotics could help against pathogens [40]. In zebrafish, *B. amyloliquefaciens* R8 administration induced a significant increase of gut xylanase activity in respect to fish fed a control diet. At the hepatic level, mRNA expression of glycolysis-related and anti-apoptotic genes was regulated; this occurred concomitantly to an increase of 3-hydroxyacyl-coenzyme A dehydrogenase and citrate synthase enzyme activities, responsible for fatty acid β-oxidation and mitochondrial integrity [64]. In addition, treating zebrafish with probiotics significantly increased disease resistance against *A. hydrophila* and *S. agalactiae*. This evidence strongly supports the idea that the administration of xylanase-expressing *B. amyloliquefaciens* R8 can improve stress response, increasing immunity and disease resistance against pathogen challenges [64]. In *X. hellerii*, diet supplementation with *B. subtilis* significantly improved growth by increasing feed assimilation and improving metabolism [52]. Inclusion of *B. subtilis* in *P. latipinna* diet, significantly improved growth and disease resistance against *A. hydrophila*, and led to a higher survival rate than in control or antibiotic-treated groups [72]. The administration of a commercial probiotic, containing *B. subtilis* and *B. licheniformis* to goldfish, *C. auratus*, significantly improved the survival rate, food digestibility, stress resistance, and immune response [55]. These two probiotic strains enhanced immune parameters, increased total protein, albumin, total globulin, lysozyme and hemolytic complement activity levels compared to control when sprayed on a dry diet and administered to tinfoil barb, *Barbonymus schwanenfeldii*. In addition, the increase in peroxidase and trypsin levels suggested a possible activation of leucocyte phagocyte activity as well as an increased resistance to pathogens and strongly advises their use against bacterial challenges [73]. The probiotic strain *P. acidilactici* was co-administered with fish oil to green terror, *A. rivulatus*, and the effects on the innate immune parameters were measured. Compared to a control diet, the experimental fish displayed a significant increase in non-specific immune system biomarkers, suggesting the positive effects of this probiotic administration [76]. A preliminary study on *P. scalare* also revealed that *E.faecium* could be used to improve the viability of this species [78]. Dietary administration of *E. cloacae* with a 2% mannan oligosaccharide (MOS), significantly increased blood cell counts and respiratory activity in the Kenyi cichlid, *Maylandia lombardoi*, against *Plesiomonas shigelloides*, suggesting the use of this probiotic strain to prevent this infection in ornamental fish aquaculture [79]. 

When adult zebrafish were fed a diet supplemented with *C. aquaticum*, a “potential-candidate” probiotic isolated from lake water samples, a set of protease and xylanase and a bacteriocin-like substance, able to counteract the negative effects of diverse pathogens, including aquatic, foodborne and plant pathogens, were produced. In fish receiving the probiotic, carbohydrate and immune-related gene expression were upregulated. Additionally, dietary probiotics increased disease resistance against *A. hydrophila* and *S. iniae*. These results clearly showed that *C. aquaticum*, as a probiotic, not only improved nutrient metabolism but also increased innate immune parameters as well as resistance against pathogens [66]. In the same fish species, administration of *Actinobacteria phylum*, *B. infantis* and *B. longum* decreased the number of gut pathogenic species. Despite this positive evidence, one week after the end of the trial, gut microflora was not significantly colonized by these two species, suggesting that these probiotics should be administered longer or should be supplied with other beneficial species for a more stable result [62]. The administration of a commercial probiotic mixture composed of *S. thermophilus* DSM 24731, *B. longum* DSM 24736, *B. breve* DSM 24732, *B. infantis* DSM 24737, *L. acidophilus* DSM 24735, *L. plantarum* DSM 24730, *L. paracasei* DSM 24733, and *L. delbrueckii *sp.* bulgaricus* DSM 24734 potentiates immune cell function, by inducing gene expression of Toll-like receptors (TLR) and other immune factors [65]. A decrease in the number of apoptotic cells was observed in the gut, suggesting the capacity of these bacteria to control immune response and inflammation [65]. In the black molly, *P. sphenops*, when challenged with *V. anguillarum*, the proteobacteria probiotic strain *Phaeobacter inhibens* S4Sm colonized the gut, causing changes in the fish microbiome able to contrast the negative impact of antibiotic treatment, suggesting that its use in aquaculture could protect fish from external pathogens [61]. 

The administration of *Thermocyclops decipiens* enriched with *B. infantis* significantly changed gut microflora composition in *P. conchonius* post-larvae [57]. Dietary protein supplement with a multispecies bacteria formulation made of several probiotic strains *B. bacterium*, *S. silivarius*, *E. faecium*, *A. oryzae*, and *Candida pintolopesii* exerted positive effects on hematological factors of the Oscar, *A. ocellatus* fingerlings, suggesting positive outcomes against pathogens in both commercial and ornamental fish trade and opens new possibility for further research [80]. 

Bacteria can also be used as a source of proteins. In a trial with *X. hellerii*, a set of artificial diets were prepared by substituting fish meal with different proportions of microbial single-cell proteins (SCPs) extracted from the gut. SCPs could be bacterial cells, either *Micrococcus* or *Bacillus*, *Azobacter* or *Streptomyces*-a- and -b-enriched diets, that induced a significant improvement of food conversion efficiency and conversion rate and a higher protein content in *X. hellerii*, thus representing a promising area of research [50]. Results presented are summarized in Table 5. 

So far, several yeast species have been used in ornamental fish culture and the positive effects of their use have been demonstrated. Molly, *P. latipinna*, fed on artemia enriched with *S. cerevisiae* cell wall, showed an improvement of reproduction, stress response, and resistance against *A. hydrophila* [81]. Similarly, a feeding trial was carried out to study the effects of *S. cerevisiae* in juvenile *A. percula* and the results showed that the treatment increased hematological and serum values (markers of the non-specific immune parameters) improving defense against *Streptococcus* sp. [82]. Diet supplementation with RNA extracted from *Kluyveromyces fragilis* significantly modulated the stress response during zebrafish early larval development, suggesting an anti-inflammatory action of RNA yeast extract [69]. Two non-*Saccharomyces* species, *Yarrowia lipolytica* 242 (Yl242) and *Debaryomyces hansenii* 97 (Dh97), significantly improved the immune system of zebrafish larvae and defense against *V. anguillarum* when administered with the diet. Treated fish showed a downregulation of immune-related genes (*IL-1β*, *TNF-α*, *IL-10*, *C3*, *MPx*), suggesting that this yeast could be used against pathogens [70]. Since a healthier status was already described in several aquaculture species including catfish, *Clarias gariepinus* [93], salmon, *Salmo salar*, [94], and trout, *Oncorhynchus mykiss* [95], the results strongly suggest the use of yeast also in ornamental aquaculture. 

## 4. Effects of Microbial Feed Additives on Ornamental Fish Growth 

The main target of aquaculture practice is to acquire the most rapid growth and the lowest production cost. To achieve this goal, several means have been established to boost the growth rate and feed consumption by adding functional feed additives [96,97]. Probiotics are among those functional feed additives showing strong effects on growth, health, and well-being. In aquaculture, investigations on probiotic-containing diets demonstrated the role of these favorable bacteria in improving gut microflora balance and in the production of extracellular enzymes able to enhance feed utilization and increase growth performance [98,99]. Probiotics can increase the uptake of nutrients, the assimilation capacity, the feed conversion ratio and improve digestibility [99,100,101]. In addition, probiotics have been proven to promote the absorption of feed through the production of extracellular digestive enzymes, i.e., amylases, proteases and lipases or intestinal alterations, resulting in a better growth [25,102,103,104,105].

Ahmadifard et al. [72] indicated that dietary inclusion of *B. subtilis* in diets of *P. latipinna* significantly improved growth and reproductive performance. It has been further reported that probiotics colonized in the gastrointestinal tract could stimulate broodstock and larvae nutrition by synthesizing the necessary nutrients and enzymes, such as proteins, vital fatty acids, and amylase, as well as protease and lipase [106]. In addition, probiotics in fish gut promoted enzyme excretion in the host by inducing maturation of the gut secretory cells [99,101]. These enzymes improved the digestion efficiency of complicated proteins and lipids included in the diet that can per se affect the assimilation rate. Similarly, He et al. [107] disclosed that the inclusion of *B. subtilis* in the diet significantly improved Koi carp, *C. carpio*, weight gain and feed conversion ratio. However, dietary probiotics had no effect on fish body skin coloration. 

The supplementation of *Lactobacillus* in black swordtail, *X. hellerii*, resulted in the promotion of growth and survival rates [48]. Significantly improved growth performance can be due to increased digestive enzymes and appetite, vitamin production, degradation of undigested elements and potential enhancement in intestinal morphology [108] (Table 6). However, dietary administration of *L. acidophilus* did not have any significant effect in goldfish, *C. auratus gibelio*, growth performance and feed utilization [109]. The inconsistent nature of these findings can be attributable to a species-specificity, stage of life, dosage, and trial condition and suggests the need for different probiotics to be assessed on desired species [110]. *P. acidilactici* has also been used in green terror, *A. rivulatus* [76], Oscar, *A. ocellatus* [111], and convict cichlid, *Amatitlania nigrofasciata* [112]. The results suggested that its dietary administration significantly improved growth efficiency and survival rate. Dietary incorporation of two *Bacillus* probiotics (*B. subtilis* and *B. licheniformis*) significantly increased growth performance and feed utilization in goldfish, *C. auratus* [55]. *Bacillus* sp. are dominant in the gastrointestinal tract of fish and shellfish [105], which are able to produce several amino acids [28] and vitamins (K and B12) [113] to boost the host’s growth performance. Similarly, the combination of *L. plantarum*, *L. delbrueckii*, *L. acidophilus*, *L. rhamnosus*, *B. bifidum*, *S. silivarius*, *E. faecium*, *A. oryzae*, and *C. pintolopesii* significantly improved the growth rate, but decreased food conversion rate (FCR) in Oscar, *A. ocellatus* [80]. Dhanaraj et al. [114] found that dietary administration of *L. acidophilus* and *S. cervisiae* showed a higher growth rate and FCR in koi carp, *C. carpio*, compared to the control. In addition, β-glucan, found in the yeast cell wall, was capable of improving immunity, growth performance, and resistance against pathogens [115]. It is likely that dietary *Lactobacillus* sp., *Azotobacter* sp., *Clostridia*, sp., *Enterbacter* sp., *Agrobacterium* sp., *Erwinia* sp., and *Pseudomonas* sp. significantly improved goldfish growth performance [116]. This mixture could increase the fermentation rate of feeds in fish intestine, and increase the absorption rate of nutrition in the digestive system [33]. In contrast, Abraham and collaborators [49] indicated that dietary inclusion of *L. sporogenes*, *L. acidophilus*, *B. subtilis*, *B. licheniformis*, *S. faecium*, and *S. cerevisiae* displayed no effect on the growth performance and feed utilization of *C. auratus* and *X. hellerii*. Similarly, dietary inclusion of *B. licheniformis*, *B. latrospore*, and *S. cerevisiae* did not make a difference in weight gain, the specific growth rate, and the feed conversion ratio in guppy, *P. reticulata* [117].

## 5. Effects of Microbial Feed Additives on Ornamental Fish Reproduction 

The beneficial effect of probiotics in reproduction was demonstrated, thanks to their ability to produce vitamin B and certain unknown stimulants [118], which in turn could play a vital role in increasing the reproduction rate of the host [126]. One example is represented by *B. subtilis*, which is able to synthesize vitamins B1 and B12, responsible for the reduction of the number of abnormal and dead larvae [51]. The effects of one year of *B. subtilis* dietary supplementation were evaluated in five ornamental fish species—*Cirrhinus mrigala*, *P. reticulata*, *P. sphenops*, *X. helleri* and *X. maculatus*—and the results obtained highlighted better reproductive performances, as witnessed by the increase in the gonadosomatic index (GSI), fecundity and fertility rate in all species analyzed. In addition, fries presented higher survival rates as well as decreases of deformities [52]. Similarly, an Artemia diet enriched with *B. subtilis* significantly improved the reproductive performance of *P. latipinna*, in terms of fry production and survival [72]. The administration of *L. rhamnosus* strongly improved zebrafish reproductive performance, acting on both fertility and fecundity [26,42]. Probiotics acted at the gonadal level by inducing follicle maturation [43,67]. A similar effect of *L. rhamnosus* was also evidenced in killifish (*Fundulus heteroclitus*) [127].

The positive effects of probiotics on male reproductive performance were first described in zebrafish by Valcarce, et al. [128], who reported an improvement of sperm quality and reproductive behavior in fish treated with *L. rhamnosus* and *B. longum* [128]. In this last model, similar results were reported by other authors, using two different *Lactobacillus* strains, *L. rhamnosus CIC 6141* and *L. casei BL23* [68]. Dietary administration of *P. acidilactici* (0.2%) and nucleotide (0.5%) positively affected sperm quality, motility and density in goldfish, *C. auratus* [129]. 

A novel aspect in this field is represented by the ability of probiotics to contrast endocrine disruptor reproductive toxicity, as observed in zebrafish exposed to bisphenol A. Probiotic co-administration, indeed, mitigates bisphenol A reproductive toxicity in zebrafish [130].

These results are summarized in Table 5.

## 6. Role of Microbial Feed Additives in Maintaining Good Water Quality of Ornamental Fish Holding Systems

Administration of probiotics in culture water can offer an advantage at any point in the species life cycle. This is of high importance, especially during larval stages, when their use can improve health conditions [131]. Probiotics in water can proliferate using available substrates and competitively exclude the pathogenic bacteria [132].

It has been suggested that water probiotics (*B. acidophilus*, *B. Subtilis*, *B. licheniformis*, *Nitrobacter* sp., *Aerobacter* sp., and *S. cerevisiae*) beneficially affect water quality through enhancing organic matter decomposition of the undesirable organic substances [133], increasing the population of food organisms, reducing pathogenic bacteria [134] and nitrogen and phosphorus concentrations and controlling ammonia, nitrite, hydrogen methane, etc., levels [135,136,137,138]. Considering that fish feed and waste are two significant parameters of the aquaculture ecological footprint, it can be argued that probiotics can contribute to reduce the environmental impact of aquaculture [139].

## 7. Current Knowledge Regarding Prebiotic and Synbiotic Use in Ornamental Fish 

Prebiotics, as promising immunostimulants, have been introduced to aquaculture as a preventive action [140]. They mainly consist of oligosaccharides such as fructooligosaccharide (FOS), galactooligosaccharide (GOS), mannan-oligosaccharide (MOS), and xylooligosaccharide (XOS), which have been proven to promote beneficial bacterial growth within the gastrointestinal tract [141,142]. Many prebiotic compounds such as FOS, GOS, MOS, inulin or β -glucan have been used in the ornamental fish culture industry (Table 7) and extensively employed as immunostimulants to improve growth performance, modulate microbial activities of the digestive tract, stimulate the immune system and enhance stress resistance. In the literature, data about the effect of prebiotic administration on growth performance in ornamental fish are reported. GOS administration was effective in gibel carp, *C. auratus gibelio* [110], and zebrafish, *D. rerio* [143]. MOS administration did not affect Siamese fighting fish, *B. splendens* [144], and clownfish, *A. ocellaris* [145] survival and growth, but positively acted on zebrafish, *D. rerio.* [146] and regal peacock cichlid, *A. stuartgranti* [147]. Focusing on XOS, it was effective on Oscar, *A. ocellatus* [148], and crucian carp, *C. auratus gibelio* [149] (Table 7 and Table 8).

The prebiotic type, dose, duration of treatment as well as host gut microbiota are the main reasons for observing contradictory results.

Dietary supplementation of prebiotic such as β-glucan [140] in koi, *C. carpio koi*, and ferula, [153] in zebrafish, GOS in goldfish, *C. auratus gibelo* [110], and zebrafish, *D. rerio* [143] increased humoral (alkaline phosphatase, lysozyme, immunoglobulin, alternative complement, and superoxide dismutase activity) and cellular (phagocytic capacity and respiratory burst activity) immune responses. Iger and Abraham [161] reported that alkaline phosphatase possesses antibacterial properties due to hydrolytic activity and its increase suggested an improved immune response. 

Nutritional supplements combining probiotics and prebiotics in the form of synergism is known as symbiotic, resulting in a more beneficial effect when compared with individual administration [162]. In a trial, angelfish, *P. scalare*, were fed with three different diets—a control diet, a diet enriched with *P. acidilactici*, and a diet enriched with *P. acidilactici* and fructooligosaccharide (FOS)—and were then exposed to environmental stress (temperature and salinity stress). Results demonstrated that fish fed *P. acidilactici* and FOS presented higher levels of lysozyme activity, immunoglobulin, and protease measured in skin mucus, in respect to *P. acidilactici* alone, suggesting the beneficial and synergic role of FOS to potentiate the probiotic effects [77]. 

In addition, in koi, *C. carpio koi*, under dietary COS combined with *B. coagulans* [140], or in rockfish, *Sebastes schlegeli*, under dietary GOS combined with *P. acidilactici* [163] or in Japanese flounder, *P. olivaceus*, dietary MOS combined with *B. clausii* [160], a significant improvement of growth performance and immune response was observed. Since synbiotic treatment has been suggested as a suitable alternative technique for pathogen prevention, their effectiveness in terms of defense against infectious diseases could be evaluated by a challenge test. Challenge tests using *A. veronii* and *Edwardsiella tarda* as pathogens were conducted in koi, *C. carpio koi* [140], and rockfish, *Sebastes schlegeli* [163], following a dietary synbiotic administration.Post-challenge mortality of fish was significantly higher in the control group than in the synbiotic group, which showed COS + *B. coagulans* and GOS + *P. acidilactici* to have a synergistic effect (Table 8). 

## 8. Research Gaps and Future Perspectives

The use of probiotics, prebiotics and synbiotics offers viable alternatives for a new generation of a higher-quality live products in terms of size, health, safety, and production time. Several studies on probiotics suggested the many advantages and benefits of their use in terms of growth performance, water quality and immune system. Despite the extensive research attempts to increase disease resistance of cultured fish by using dietary probiotics, prebiotics, and synbiotics, a limited number of studies were so far conducted on ornamental fish. In the near future, the application of prebiotics and synbiotics will be pivotalfor the development of a sustainable ornamental fish production. However, there is still a need to understand the mechanisms of action of pre-, pro- and synbiotics on both gastrointestinal health and animal welfare. Further studies must be undertaken to determine the composition of microbial communities and their administration as live feed additives. They are, indeed, temperature sensitive and can be killed by the high temperature during pelleting procedures, thus great efforts have been made to fill this gap, resulting their microencapsulation, freezing and inclusion in protective matrices, promising strategies to increase microbe viability and survival in feed [164]. Additionally, detailed investigations about the time, type, frequency and dose of live feed additive application must be conducted. Considering the promising immunomodulatory effects of these functional feed additives, their application can also be taken into account in ornamental fish larviculture. 

## Figures and Tables

**Table 1:** Common parasites isolated from ornamental fishes.

Scientific Name	Common Name	Occurrence	Reason	Host
*Ichthyobodo necator*	Flagellates (Costia)	Skin, gills	Poor water quality	*Carassius auratus*
*Spironucleus vortens*	Binucleate flagellate	Gastrointestinal tracts	Poor water quality, malnutrition, overcrowding, fluctuating temperatures	*Pterophyllum* sp.
*Cryptobia lubilans*	Cryptobia	Gastrointestinal tracts, abdominal organs	Poor water quality	*Carassius auratus*, *Herichthys yanoguttatus*, *Cichlasoma cyanoguttatum*, *Cichlasoma meeki*
*Amyloodinium ocellatum*	Dinoflagellates	Gill, fins, skin	Poor water quality	*Amphiprion* sp.
*Oodinium pilluarius*	Dinoflagellates	Gill, skin	Poor water quality	*Carassius auratus*
*Ichthyophthirius multifilis*	Holotrichous ciliate	Gill, eyes, skin	Poor water quality	*Carassius auratus*
*Cryptocaryon irritans*	Marine white spot (Ich)	Gill, skin	Poor water quality	*Carassius auratus*, *Zebrasoma xanthurum*, *Chaetodon adiergastos*, *Paracanthurus hepatus*
*Trichodina* sp.	Monogenean	Gill, skin	Poor water quality	*Carassius auratus*, *Betta splendens*, *Pterophyllum* sp., *Paracheirodon innesi*, *Trichopodus trichopterus*, *Xiphophorus hellerii*, *Betta splendens*
*Trichodonella* sp.	Monogenean	Gill, skin	Poor water quality	*Carassius auratus*, *Betta splendens*, *Pterophyllum* sp., *Paracheirodon innesi*, *Trichopodus trichopterus*
*Tripartiella* sp.	Monogenean	Gill, skin	Poor water quality	*Xiphophorus hellerii*, *Betta splendens*
*Epistylis* (*Heteropolaria*) sp.	Monogenean	Skin, fins, gills	High organic content waters	*Astronotus ocellatus*, *Carassius auratus*, *Poecilia reticulata*, *Cichlasoma nigrofasciatum*, *Pterophyllum scalare*, *Poecilia sphenops*
*Tricodina* sp.	Monogenean	Gill, skin	Poor water quality	*Carassius auratus*, *Poecilia reticulata*, *Pterophyllum scalare*, *Symphsodon discus*, *Poecilia latipinna*
*Chilodonella*	Ciliate	Gill, skin	Poor water quality	*Astronotus ocellatus*, *Carassius auratus*, *Poecilia reticulata*, *Cichlasoma nigrofasciatum*, *Pterophyllum scalare*, *Poecilia sphenops*
*Hexamita*	Flagellate	Gastrointestinal duct	Poor water quality	*Poecilia reticulata*, *Pterophyllum scalare*
*Dactylogyrus* sp.	Monogenean	Skin, fins, gills	Poor water quality	*Carassius auratus*
*Gyrodactylus* sp.	Monogenean	Skin, fins, gills	Poor water quality	*Carassius auratus*
*Bothriocephalus* sp.	Tapeworms	Digestive tract, coelomic cavity	Poor water quality	*Xiphophorus hellerii*
*Eustrongylides* sp.	Nematode	Muscle, liver, intestinal, Abdomens, guts	Poor water quality	*Poecilia reticulata*, *Danio rerio*, *Pterophyllum scalare*
*Capillaria pterophylli*	Nematode	Intestinal tract	Poor water quality	*Archocentrus nigrofasciatus*, *Pelvicachromis pulcher*, *Carassius auratus*, *Trichogaster trichopterus*, *Hyphessobrycon anisitsi*
*Pentastomids*	Tongue worms	Skin, body cavity, connective tissue, muscle	Poor water quality	*Archocentrus nigrofasciatus*, *Xiphophorus hellerii*, *Poecilia sphenops*, *Xiphophorus maculatus*
*Argulus*	Fish louse	Skin	Poor water quality	*Carassius auratus*
*Lernaea*	Anchor worm	Skin	Poor water quality	*Carassius auratus*

**Table 2:** Common bacteria isolated from ornamental fishes.

Scientific Name	Common Name	Symptoms	Reason	Host
*Aeromonas hydrophila*	*Aeromonas* Septicemia (MAS)	Hemorrhagic septicemia in skin, fin, oral cavity, ulceration in epidermis	Stress, overcrowding, contaminated water	*Carassius auratus*, *Xiphophorus hellerii*, *Colisa lalia*, *Molliensia sphenops*
*Aeromonas caviae*	Gastroenteritis	Hemorrhagic septicemia	Contaminated water	*Carassius auratus*, *Datnioides polota*, *Poecilia sphenops*, *Xiphophorus hellerii*, *Carassius auratus*, *Datnioides polota*, *Poecilia sphenops*
*Aeromonas salmonicida*	Furunculosis	Hemorrhage in skin, fin, oral cavity, muscles	Contaminated water	*Carassius auratus*, *Puntius conchonius*
*Flexibactor columnar*	Columnaris	Skin, gills lesions and necrosis	Crowded unhygienic conditions, cold snaps stress	*Carassius auratus*, *Paracheirodon innesi*
*Streptococci iniae*	Streptocosis	Erratic swimming, darkening of skin, hemorrhages in eye, gill, vent, ascites, dropsy,	Contaminated water	*Pethia conchonius*, *Danio rerio*
*Edwardsiella ictaluri*	*Edwardsiellosis*	Ulcer in skin, spiral and erratic swimming, hemorrhage and inflammation in tissues	Contaminated water	*Danio devario*, *Puntius conchonius*, *Molliensia sphenops*
*Vibrio vulnificus*	Vibriosis	Erratic swimming, hemorrhage	Contaminated water	*Poecilia sphenops*

**Table 3:** Common virus isolated from ornamental fishes.

Name	Symptoms	Host
Carp pox viral disease (CYHV—1)	Grey lesions on the body and fins	*Carassius auratus*
Cyprinid herpesviral disease (CYHV—2)	Hematopoietic necrosis	*Carassius auratus*
Herpesviral hematopoietic necrosis (HVHN/CyHV-2)	Softening and discoloration of the spleen and kidney, necrotic foci in the hematopoietic tissue, splenic pulp, pancreas	*Carassius auratus*
Spring Viremia of Carp (SVC)	Darkening of the skin, exophthalmia, ascites, pale gills, hemorrhage and a protruding vent with thick mucoid fecal casts	*Carassius auratu*
Viral hemorrhagic septicemia (VHS)	lethargy, darkening of the skin, exophthalmia, anemia (pale gills), hemorrhages at the base of the fins, gills, eyes, skin	*Pterophyllum* *scalare*
Banggai Cardinalfish Iridovirus (BCIR)	Lethargy. respiratory distress (rapid movement of opercula)	*Barbus graellsii*
Dwarf Gourami Iridovirus (DGIR)	Necrosis in spleen and kidney, pale coloration, loss of appetite, lesions on the body ascites (abdominal swelling)	*Pterapogon kauderni*
Reovirus (head and lateral line erosion, HLLE)	Hemorrhagic	*Trichogaster lalius*
Koi herpesviral disease (KHV)	Necrosis of gill epithelium copious secretion of mucus on the gills and skins and necrosis of gill tissue, lethargy, anorexia, excessive gill necrosis, gill and body mucus and signs such as ulceration, skin hemorrhages, petechial ecchymosis and fin rot, erosion of primary lamellae, fusion of secondary lamellae and swelling at the tips of the primary and secondary lamellae	*Pterophyllum* sp., *Pterophyllum scalare*
Iridovirus	Abdominal distention exophthalmia and pale gills abdominal swellings	*Poecilia reticulata*, *Osphronemus goramy*, *Pterophyllum* sp.

**Table 4:** Common fungi isolated from ornamental fishes.

Scientific Name	Common Name	Condition	Host
*Saprolegnia* sp.	Saprolegniasis	Poor water quality	*Pterophyllum* sp.,
*Icthyophonus hoferi*	*Ichthyophoniasis*	Skin a sandpaper texture	*Gymnocorymbus ternetzi*, *Pentius tetrazona*
*Aphanomyces invadans*	Epizootic ulcerative syndrome (EUS)	Erode underlying tissues, unilateral or bilateral clouding of the eye, red spots or small hemorrhagic lesions on the surface of fish, ulcers and eventually large necrotic erosions	*Barbus thamalakanensis*, *Glossogobius giuris*, *Carassius auratus*

**Table 5:** The effects of microbial feed additives on ornamental fish health, welfare and reproduction.

Species	Probiotic Strain	Effects	References
Siamese fighting fish (*B. splendens*)	*L. plantarum KKU CRIT5*	Lack of evident beneficial effects. Timing and dose of administration are under review	[47]
Green swordtail (*X. helleri*)	*L. acidophilus*	Positively modulated mucosal immune parameters	[48]
	Commercial formulation (*Lactobacillus* sp., *Bacillus* sp., *S. faecium* and *S. cereviasiae*)	No beneficial effect observed against bacterial challenges	[49]
	*Streptomyces* sp.	Improves food conversion efficiency and conversion rate	[50]
	*B. subtilis*	Increases the GSI, fertility and fecundity. Decreases morphological alteration of the larvae	[51,52]
	*B. subtilis*	Increases the GSI, fertility and fecundity. Decreases morphological alteration of the larvae	[51,52,53]
Platy fish (*X. maculatus*)	Commercial formulation containing *Lactobacillus* sp.	Increase of muscle mass due to protein increase and fat decrease	[54]
Goldfish (*C. auratus*)	Commercial formulation (*Lactobacillus* sp., *Bacillus* sp., *S. faecium* and *S. cereviasiae*)	No beneficial effect observed against bacterial challenges	[49]
	Mix of *Lactobacillus* sp. and *Bacillus* sp.	No beneficial effect observed against *Pseudomonas fuorescens*	[49]
	Commercial mix (*B. subtilis* and *B. licheniformis*)	Improvement of food digestibility, stress resistance and immune response	[55]
	*L. casei*	Faster recovery after the air-dive/stress test	[56]
Porthole livebearer (*P. gracilis*)	*B. coagulans (L. sporogens*), *B. mesentericus*	Scarce colonization of the gut but induction of significant microflora composition	[57]
Rosy barb (*P. conchonius*)	mixture of *Bacillus* sp. and *Lactobacillus* sp.	Decreases mortality due to improvement in the quality of rearing waters	[58]
Giant gourami (*O. goramy*)	*L. fermentum (KT183369) and B. subtilis *sp.* inaquasporium (KR816099*)	Good capacity to colonize the host gut	[59,60]
Black molly (*P. sphenops*)	*B. pumilus RI06-95Sm*	Gut colonization; increases protection against pathogens	[61]
	*P. inhibens S4Sm*	Increases tolerance to antibiotic treatment	[61]
	*L. rhamnosus*	Decreases the number of gut pathogenic CFU/mL	[62]
*Zebrafish* (*D. rerio*)	*L. rhamnosus*	Skeletogenesis acceleration	[38]
	*L. rhamnosus*	Affects lipid and vitamin D metabolism, with a positive role in backbone calcification	[63]
	*B. amyloliquefaciens R8*	Increases xylanase activity, 3-hydroxyacyl-coenzyme A dehydrogenase and citrate synthase. Increases mRNA of glycolysis-related and anti-apoptotic signals	[64]
	Commercial mixture (*S. thermophilus DSM 24731*, *B. longum DSM 24736*, *B. breve DSM 24732*, *B. infantis DSM 24737*, *L. acidophilus DSM 24735*, *L. plantarum DSM 24730*, *L. paracasei DSM 24733*, *L. delbrueckii *sp.* bulgaricus DSM 24734*),	Increased expression of Toll-like receptors and other immune factors. Decreases number of apoptotic cells	[65]
	*L. rhamnosus* IMC 501	Upregulation of genes involved in innate immune responses and decrease in the abundance of stress- and apoptotic-related genes	[40]
	*B. infantis and B. longum*	Decreased number of pathogenic species, but scarce gut colonization	[62]
	*C. aquaticum*	Increased hepatic mRNA expression of carbohydrate metabolism-related- and Innate immune-related genes	[66]
	*L. rhamnosus*	Increased fertility, fecundity and follicle maturation	[26,42,67]
	*L. rhamnosus CIC6141* *L. casei BL23*	Improved reproduction	[68]
	*K. fragilis*	Decreases stress biomarkers	[69]
	*Yarrowia lipolytica* 242 (Yl242) and *Debaromyces hansenii* 97 (Dh97)	Improved the immune system (downregulation of *1b*, *tnfa*, *il10*, *c3*, *mpx*)	[70]
	*L. rhamnosus*	Positive modulation of signal involved in lipid and vitamin D metabolism	[71]
*Clownfish* (*A. ocellaris*)	*B. subtilis*	Higher survival in antibiotic-treated fish, increased fertility and fecundity	[72]
Sailfin Molly (*P. latipinna*)	*B. subtilis* and *B. licheniformis*	Increase in peroxidase and trypsin levels and resistance against bacterial challenges	[73]
Tinfoil barb (*B. schwanenfeldii*)	Commercial mixture (*B. subtilis*, *B. licheniformes*, *L. acidophilus* and *S. cerevisiae*)	Improved water quality by reducing metabolic waste and stress response	[74]
Marbled hatchetfish (*C. strigata*)	Commercial mixture (*B. subtilis*, *B. licheniformes*, *L. acidophilus and S. cerevisiae*)	Improved water quality by reducing metabolic waste and stress response	[75]
Cardinal tetra (*P. axelrodi*)	*P. acidilactici*	Significant increase in all non-specific immune system biomarkers (lysozyme activity, total immunoglobulin and alternative complement activity)	[76]
Green terror (*A. rivulatus*)	*P. acidilactici*	Improved stress response (modulation of lysozyme activity, immunoglobulin and protease levels)	[77]
Angelfish (*P. danio*)			
*Angelfish* (*P. scalare*)	*E. faecium*	Improved fish viability	[78]
	*E. cloacae*	Improved resistance against *P.shigelloides* challenge (increased blood cell counts and respiratory activity)	[79]
Kenyi cichlid (*M. lombardoi*)	*B. infantis*	Alteration of gut microbiota composition	[57]
Rosy barb (*P. conchonius*)	Commercial mixture (*Lactobacillus* sp, *B. bacterium*, *S. silivarius*, *E. faecium*, *A. oryzae*, *C. pintolopesii*	Positive effects on hematological factors	[80]
Oscar (*A. ocellatus*)	*B. subtilis*	Increase in the GSI, fertility and fecundity. Decreased morphological alteration of the larvae	[51]
Guppy (*P. reticulata*)	*B. subtilis*	Increase in the GSI, fertility and fecundity. Decreased morphological alteration of the larvae	[51]
Gold black molly (*P. sphenops*)	*S. cerevisiae*	Improvement of reproduction, stress response and resistance against pathogens	[81]
Sailfish molly (*P. latipinna*)	*S. cerevisiae*	Improvement of growth performance, feed utilization and disease resistance	[82]
Orange clownfish (*A. percula*)			

**Table 6:** Effect of microbial feed additive supplementation on ornamental fish growth performance and survival rate.

Probiotic Strain	Fish Name	Parameters	Reference
Probiotic preparation A *	*Astronotus ocellatus*	SGR	[80]
*L. acidophilus* (LAD) and/or brewer’s yeast	*Cyprinus carpio koi*	SGR	[114]
*Lactobacillus* sp.	*Xiphophorus hellerii*	SGR	[118]
Probiotic preparation B **	*Carassius auratus*	SGR, SR	[116]
*S. cerevisiae*	*Cichlasoma trimaculatum*	SGR, SR	[119]
*Streptomyces*	*Xiphophorus helleri*	SGR, AGR, RGR, FCR	[50]
Commercial probiotics	*Carassius auratus*	SGR, WG, SR, FCR	[55]
*S. cerevisiae*	*Carassius auratus*	SGR, WG, FCR, PER	[120]
*Pseudomonas* sp.	*Carassius auratus*	SGR, FCR, RGR	[121]
*B. cereus*	*Trichogaster trichopterus*	SGR, SR	[122]
*Lactobacillus*	*Carassius auratus*	SGR, SR	[54]
*Bacillus*, *S. cerevisiae*	*Poecilia reticulata*	SR	[117]
Probiotic preparation C ***	*Poecilia sphenops*	SR	[123]
*L. sporogenes*	*Carassius auratus*	WG	[124]
*L. helveticus*	*Carassius auratus*	SGR	[125]

AGR: absolute growth rate, SGR: specific growth rate, RGR: relative growth rate, FCR: feed conversion ratio, SR: survival rate, WG: weight gain, RGR: relative growth rate, FCE: feed conversion efficiency, and RGR: relative growth rate; * contains *L. paracasei*, *L. rhamnosus*, *L. acidophilus*, *L. bulgaricus*, *B. breve*, *B. infantis* and *S. thermophilus*; ** contains (*Lactobacillus* sp., *Azotobacter* sp., *Clostridia* sp., *Enterbacter* sp., *Agrobacterium* sp., *Erwinia* sp., *Pseudomonas* sp. and lactate formed bacteria); *** contains a combination of *S. faecalis*, *C. butyricum*, *B.mesentericus* and *L. sporogenes*.

**Table 7:** The effects of different prebiotic substances on ornamental fish species.

Prebiotic Substances	Target Species	Measured Responses	References
MOS	*Betta splendens*	GP, IR↑, IHM	[144]
	*Danio rerio*	GP↑, SR↑	[146]
	*Aulonocara stuartgranti*	GP↑, BC, DEA↑, IM	[147]
	*Amphiprion ocellaris*	GP, SR↑	[145]
XOS	*Astronotus ocellatus*	GP↑, IM, IHM, SR↑	[148]
	*Carassius auratus*	GP↑, DEG↑, SR	[149]
β-glucan, chitosan and raffinose	*Cyprinus carpio koi*	GP↑, IR↑, DR↑	[140]
Preparation D *	*Xiphophorus maculatus Xiphophorus hellerii*	GSI↑, RP↑, SR↑	[150,151]
Inulin	*Carassius auratus*	GP↑, FU↑, BC, IM, HP, SR↑	[152]
GOS	*Carassius auratus*	GP, IR↑, SR	[110]
	*Danio rerio*	GP, IR↑	[143]
FOS	*Aulonocara stuartgranti*	GP↑, BC, DEA↑, IM	[147]
Ferula (*Ferula asafoetida*) powder	*Cyprinus carpio koi*	GP↑, FU↑, IR↑, HP↑, DEA↑, IM, DR↑, SR↑	[153]
Eryngii mushroom (*Pleurotus eryngii*)	*Cyprinus carpio koi*	GP↑, FU↑, IR↑, HP↑, DEA↑, IM, DR↑, SR↑	[154]
Preparation E **	*Carassius auratus*	GP, IR, DEA, IM, DR	[155]

Notes: * composed of β-glucans and MOS. ** composed of partially autolyzed brewer’s yeast Saccharomyces cerevisiae, dairy components, and fermentation products. Prebiotic abbreviations: FOS—fructooligosaccharide, GOS—galactooligosaccharide, MOS—mannan-oligosaccharide, and XOS—xylooligosaccharide. Parameters investigated: GP—growth performance, FU—feed utilization, BC—body composition, IR—immunological response, HP—hematological/serum biochemical parameters, DEA—digestive enzyme activity, IM—intestinal microflora, IHM—intestinal histomorphology, RP—reproductive performance, DR—disease resistance, and SR—survival rate. ↑ denote significant increase.

**Table 8:** The effects of different synbiotic mixtures on ornamental fish species.

Synbiotic (Pre/Pro)	Target Species	Measured Responses	Reference
FOS/*P. acidilactici*	*Pterophyllum scalare*	GP↑, IR↑, IM, SR↑	[78]
FOS/*E. faecium*	*Heros severus*	GP↑, FU↑, IR↑, DEA↑, SR↑	[156]
	*Carassius auratus gibelio*	GP↑, IR↑, SR	[157]
	*Danio rerio*	GP↑, FU↑, RP↑, SR↑	[158]
COS/*B. coagulans*	*Cyprinus carpio koi*	GP↑, IR↑, HP↑, DR↑, SR↑	[159]
FOS, MOS/*B. clausii*	*Paralichthys olivaceus*	GP↑, FU↑, BC, IR↑, HP, DEA↑, SR↑	[160]

Notes: Bacterial genera abbreviations: B. = Bacillus, E. = Entercoccus, and P. = Pediococcus. Prebiotic abbreviations: COS—chitosan oligosaccharides, FOS—fructooligosaccharide, GOS—galactooligosaccharide, and MOS—mannan oligosaccharide. Parameters investigated: GP—growth performance, FU—feed utilization, BC—body composition, IR—immunological response, HP—hematological/serum biochemical parameters, DEA—digestive enzyme activity, IM—intestinal microflora, RP—reproductive performance, DR—disease resistance, and SR—survival rate. ↑ denote significant increase.

## Data Availability

Data available on request due to restrictions, e.g., privacy or ethical. The data presented in this study are available on request from the corresponding author.
